# Impact of Somatosensory Training on Neural and Functional Recovery of Lower Extremity in Patients with Chronic Stroke: A Single Blind Controlled Randomized Trial

**DOI:** 10.3390/ijerph18020583

**Published:** 2021-01-12

**Authors:** Reem M. Alwhaibi, Noha F. Mahmoud, Mye A. Basheer, Hoda M. Zakaria, Mahmoud Y. Elzanaty, Walaa M. Ragab, Nisreen N. Al Awaji, Hager R. Elserougy

**Affiliations:** 1 Rehabilitation Sciences Department, Health and Rehabilitation Sciences College, Princess Nourah bint Abdulrahman University, Riyadh 11671, Saudi Arabia; rmalwhaibi@pnu.edu.sa (R.M.A.); NFMahmoud@pnu.edu.sa (N.F.M.); 2 Department of Clinical Neurophysiology, Faculty of Medicine, Cairo University, Cairo 12613, Egypt; mye.basheer@kasralainy.edu.eg; 3 Department of Neuromuscular Disorders and Its Surgery, Faculty of Physical Therapy, Cairo University, Cairo 12613, Egypt; dr.hodazakaria@cu.edu.eg (H.M.Z.); mahmoud.yassen@deraya.edu.eg (M.Y.E.); walaa.ragab@cu.edu.eg (W.M.R.); 4 Department of Neuromuscular Disorders and Its Surgery, Faculty of Physical Therapy, Deraya University, New Menya 11159, Egypt; 5 Department of Physical Therapy, Faculty of Medical Rehabilitation Sciences, Taibah University, Medina 42353, Saudi Arabia; 6 Health Communication Sciences Department, College of Health and Rehabilitation Sciences College, Princess Nourah Bint Abdulrahman University, Riyadh 11671, Saudi Arabia; NNAlAwaji@pnu.edu.sa; 7 Department of Neuromuscular Disorders and Its Surgery, Faculty of Physical Therapy, Misr University for Science and Technology, Giza 77, Egypt

**Keywords:** stroke, lower extremity, functional independence measure, quantitative electroencephalography, somatosensory training

## Abstract

Recovery of lower extremity (LE) function in chronic stroke patients is considered a barrier to community reintegration. An adequate training program is required to improve neural and functional performance of the affected LE in chronic stroke patients. The current study aimed to evaluate the effect of somatosensory rehabilitation on neural and functional recovery of LE in stroke patients. Thirty male and female patients were recruited and randomized to equal groups: control group (GI) and intervention group (GII). All patients were matched for age, duration of stroke, and degree of motor impairment of the affected LE. Both groups received standard program of physical therapy in addition to somatosensory rehabilitation for GII. The duration of treatment for both groups was eight consecutive weeks. Outcome measures used were Functional Independent Measure (FIM) and Quantitative Electroencephalography (QEEG), obtained pre- and post-treatment. A significant improvement was found in the FIM scores of the intervention group (GII), as compared to the control group (GI) (*p* < 0.001). Additionally, QEEG scores improved within the intervention group post-treatment. QEEG scores did not improve within the control group post-treatment, except for “Cz-AR”, compared to pretreatment, with no significant difference between groups. Adding somatosensory training to standard physical therapy program results in better improvement of neuromuscular control of LE function in chronic stroke patients.

## 1. Introduction

Stroke is a common cause of disability in the world [1]. Almost three-quarters of cases occur in low- to middle-income countries, leading to residual motor disabilities and intensive rehabilitation needs [2,3]. After stroke, many functions are affected, including both basic and instrumental daily living activities [2] and sensorimotor skills [4]. Lower extremity (LE) deficits are present in two-thirds of stroke patients, affecting motor control, gait, and balance, which in turn lead to poor quality of life and various degrees of dependence [5,6], which in turn require effective treatment and are considered a priority in rehabilitation [7].

Many approaches of rehabilitation are recommended to enhance motor recovery in stroke patient [8,9,10,11]; among them is the somatosensory stimulation approach, which is a new noninvasive intervention that stimulates the motor cortex through its connections with the sensory cortex, acting on the somatosensory system neuroplasticity [8,12]. It includes sensory stimulation and sensory retraining/re-education [13,14,15,16,17]. Sensory stimulation includes various methods, such as electrical stimulation (e.g., transcutaneous electrical nerve stimulation/TENS) [18], proprioceptors training, constraint-induced movement therapy (CIMT) [8], and thermal stimulation (TS) [19].

In normal individuals, TS causes greater brain area activation, compared to tactile and mechanical stimuli [20,21]. TS is a cheap and convenient technique that can be used in rehabilitation and home settings. It depends on stimulating cold and hot receptors alternatively, sending signals to the lateral spinothalamic tract through the spinal cord and up to the thalamus, to the somatosensory cortex [21]. Adding TS to the standard rehabilitation program in stroke patients resulted in significant improvement in outcome measures of both upper and lower extremities, compared to the standard rehabilitation program alone [19,22,23,24,25,26,27].

Electroencephalography (EEG) is a neurophysiological technique that measures cortical activity and brain waves. It can determine the effect of a treatment approach used in stroke patients [2]; that is why this safe technique can be of great help in detecting which treatment modality can lead to better improvement in patients’ performance. EEG has four frequency bands: delta, theta, alpha, and beta, with frequency ranges of (1–4 Hz), (4–8 Hz), (8–12 Hz), and (12–30 Hz), respectively. Brain status of the stroke patient can be characterized by Quantitative EEG (QEEG), which is very valuable for decision-making in clinical practice, as reported by many authors [28,29,30].

Despite the effectiveness of TS, only two studies were performed on LE function in chronic stroke patients: One of them measured functional outcomes, post-treatment, with no objective evaluation [26], and the other one used a different TS protocol, wherein thermal pain receptors were stimulated [25]. Because stroke recovery is a very complicated process [8,11], and multiple methods of rehabilitation techniques are used, therapists may find it difficult to decide which approach to adopt in patient management in order to achieve the greatest functional improvement with minimal cost of time and money.

This study aimed to investigate the influence of adding TS augmented with visual, auditory, and tactile somatosensory rehabilitation to standard rehabilitation on the functional recovery of LE in chronic stroke patients and how that can affect brain activity, using QEEG.

## 2. Materials and Methods

### 2.1. Study Design

The current single-blind, randomized, controlled study was approved and issued by the Faculty of Physical Therapy Ethical Committee, Cairo University, Egypt (P.T. REC/012/002153). It followed the ethical research principles of the World Medical Association (WMA) Declaration of Helsinki for human subjects. It started in January 2019 and continued until February 2020.

Forty-seven patients (*n* = 47) were recruited from the outpatient clinical units of Cairo University and assessed for eligibility; seventeen (*n* = 17) were excluded, and thirty patients (*n* = 30) were included and randomly assigned, by a random-number computer-generator, into two equal groups: control group (GI) and intervention group (GII), 15 each. Patients in both groups were kept ignorant of which type of physical-therapy treatment they were going to receive. Eligible patients were included in the study if they were diagnosed with a first-ever ischemic stroke, resulting in hemiparesis confirmed by MRI; had a stroke duration range between 6 and 18 months; reported medically stable by the treating neurologist; scored ≥ 24 on the Mini-mental state examination; scored as grade 1, 1+, or 2 on Modified Ashworth Scale (MAS) for LE; scored on Brunnstrom Recovery Stage of LE 4 or 5; and, finally, if scored as 2 for tactile sensation and stereognosis and 3 for kinesthetic sense, on Nottingham Sensory Assessment (NSA) scale. Patients were excluded from the study if they had cardiac arrhythmia, uncontrolled hypertension, obstructive pulmonary disease, any thermal application contraindication, or any previous orthopedic or neurological problem other than stroke. A proper explanation of the treatment protocol was given, and a consent form was signed by all the participants.

### 2.2. Sample Size

The sample size for this study was calculated by using the G*power program 3.1.9 (G power program version 3.1, Heinrich-Heine-University, Düsseldorf, Germany) for a one-tailed test. Sample size calculation was based on F tests (MANOVA: Special effects and interactions), Type I error (α) = 0.05, power (1-β error probability) = 0.80, Pillai V = 0.6650, and effect size f2 (V) = 0.199423, with 2 independent groups’ comparison for 4 major variable outcomes. The appropriate minimum sample size for this study was 30 patients (*n* = 15 patients in each group as a minimum).

### 2.3. Rehabilitation Program

All recruited patients received 3 treatment sessions weekly, for 8 weeks. Vital signs were measured before each session and once again after finishing the cyclic training.

- (GI) received 4 components of standard physical therapy program (cyclic training, graduated isotonic exercises, stretching exercises, and sit-to-stand functional task training) sequentially, as follows:
- Constant speed cyclic training, using a motorized cycle-ergometer (Kettler recumbent exercise bike Giro. R3, Germany) for 25 min (min) [31]. Patient’s feet were firmly strapped to the pedals. The distance between the crank axis and the seat was adjusted by allowing as far as 110°–120° of knee flexion during pedaling. Pedaling consisted of three phases: warm-up, active pedaling, and cool down. The warm-up phase was a 5 min passive constant speed cycling, at 25 rpm. Active pedaling was performed for 15 min, followed by a 5 min cool-down phase of passive cycling, with a constant speed of 25 rpm too [31,32].
- 15 min of graduated LE isotonic exercises for hip, knee, and ankle joints in various positions.
- 10 min of gentle sustained stretching for LE muscles, from supine lying position.
- 10 min of graduated sit-to-stand functional task training, starting with the patient’s feet aligned with the chair’s front legs and progressing through the sessions till the patient was able to stand by placing the affected LE backward and the non-affected forward (most difficult graduation). Any deviation during the performance of the functional activity was not allowed [33].

In (GII), patients received the same standard program of physiotherapy, with the sequence as (GI), but for a duration of 30 min, only divided into 15, 10, 5, and 5 min for each component, respectively. Patients also received thermal stimulation (TS) training augmented with tactile, visual, and auditory stimulation as follows:

Patients assumed a fully supported and relaxed supine lying position, facing a mirror adjusted for them to easily observe the LE position and movement, to augment both senses.

A careful checkup was performed for the LE before and after each session. Each patient was advised, in case of developing an uncomfortable thermal sensation, to actively move the LE away from the stimuli, if they can, or ask for help from the treating therapist.

TS was performed by using four hot pads (~75 °C, Whitehall Manufacturing, City of Industry, CA, USA) and four cold pads (~0 °C, Whitehall Manufacturing, CA, USA), each wrapped in towels, to buffer thermal conduction. To avoid ceiling duration of heating and cooling phases, the hot-pad surface temperature ranged from 46 to 47 °C and was controlled by using a digital thermometer, while the cold-pad temperature ranged from 7 to 8 °C and was applied for 30 s [22].

The session started by 15 s of hot-pad application on the affected LE, followed by 30 s of augmented training, during which each patient was instructed to try to move the affected LE actively as much as possible, using a firm command from the therapist (auditory stimulation), while tapping the moving extremity (tactile stimulation) and observing the mirror while doing the requested movement (visual stimulation). Finally, a 30 s cold application was applied, followed by a 30 s augmented exercise training of the affected LE. The previous set was repeated 10 times.

### 2.4. Clinical Evaluation

Clinical evaluation was executed before treatment and repeated after 8 weeks for each patient, using Functional Independence Measure (FIM) as a measure of functional disability. Quantitative Electroencephalogram (QEEG) was also used to measure neural recovery.

FIM includes 18 items divided to two domains: The first domain is the motor function, which includes 13 items, and the second is the cognitive function which includes 5 items. Each item can be scored from 1 to 7 or from total dependence–total independence [34,35]. In the current study, only the items concerning the LE function were measured which included transfer (bed/chair/wheelchair–toilet-bath/shower) and locomotion items (walk/wheelchair–stairs). FIM score ranged from 5 to 35 [34].

QEEG (Mizar–PC Peripheral System CE Version–B9800037800, Florence, Italy) was used to record the pattern of brain waves, by a clinical neurophysiologist, on a computer, while the patient lay comfortably, with closed eyes, to minimize artifacts from eye movements or any visual feedback. The data were recorded according to the International 10–20 system with Ag/AgCl electrodes, using a unipolar montage, while the impedance was kept constant.

The recording data were then transformed into the mapping program, to perform a spectral analysis. Data in the current study were measured from the motor area (Cz), parietal (Pz), and frontal (Fz). The equation utilized to detect brain activity was mean values of mean frequencies of [(alpha + beta)/(delta + theta)] ratio [30].

### 2.5. Statistical Analysis

In the current study, to compare sex and side of affection, the Chi-squared test was used. The Shapiro–Wilk test was used to check for data normality, while Levene’s test was used to check homogeneity between groups. To compare between patients’ characteristics in both groups, the t-test was conducted. Mixed MANOVA was used to measure changes pre- and post-treatment, in scores of FIM, Cz-AR, Pz-AR, and Fz-AR within and between groups. For subsequent multiple comparison, a post hoc test was performed, using the Bonferroni correction. The level of significance was set at *p* < 0.05. Statistical package for social studies (IBM SPSS, Chicago, IL, USA) version 25 was used for statistical analysis.

## 3. Results

**Figure 1:**
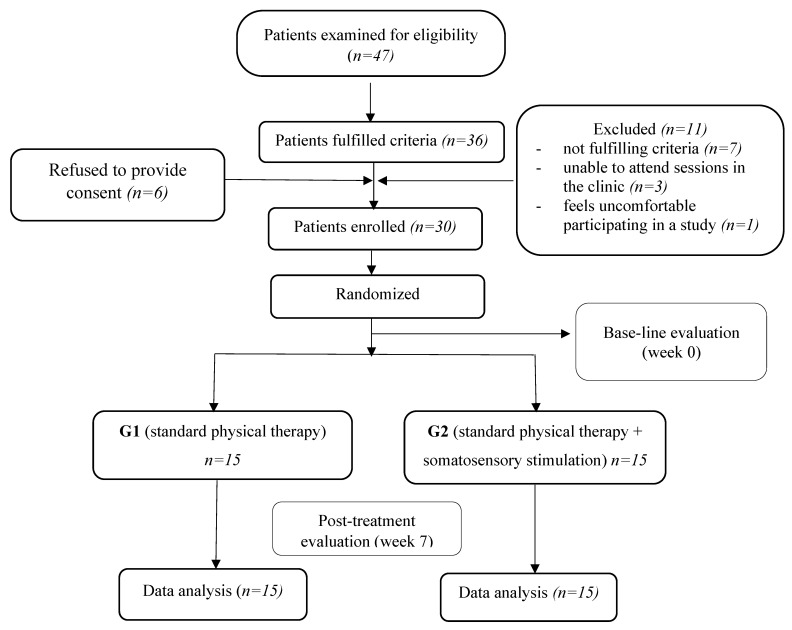
Diagram showing the flow of the study.

Forty-seven stroke patients were screened for eligibility. Eleven did not match the specified inclusion criteria of the study, six refused to provide consent, and the remaining 30 patients completed the study (Figure 1).

### 3.1. Subject Characteristics

The patient characteristics of both groups are shown in Table 1. No significant difference was found between GI and GII in the mean age, body mass index (BMI), and duration of illness (*p* > 0.05). No significant difference was found between GI and GII in gender and side of affection (*p* > 0.05).

### 3.2. Effect of Treatment on FIM, Cz-AR, Pz-AR, and Fz-AR

Mixed MANOVA showed a significant interaction between treatment and time (F = 5.97, *p* = 0.002). A significant main effect of time (F = 54.36, *p* = 0.001) with no significant main effect of treatment (F = 1.19, *p* = 0.33) was reported. A comparison between and within groups is shown in Table 2.

#### 3.2.1. Within-Group Comparison

Both groups revealed a significant increase in FIM and Cz-AR after treatment, compared with pretreatment scores (*p <* 0.001). There was a significant increase in Pz-AR and Fz-AR in GII (*p* < 0.01), while there was no significant difference in Pz-AR and Fz-AR in GI (*p* > 0.05) after treatment.

#### 3.2.2. Between-Group Comparison

No significant difference was found between both groups in all parameters of pretreatment (*p* > 0.05). Meanwhile, a significant increase in FIM in GII was found when compared with that of the control group (*p* > 0.001) after treatment. No significant difference in Cz-AR, Pz-AR, and Fz-AR between GI and GII was reported after treatment (*p* > 0.05).

## 4. Discussion

The present study investigated the influence of LE somatosensory training on functional improvement and cortical recovery of stroke patients measured by FIM and QEEG. The applied somatosensory training was an individualized thermal stimulation protocol augmented with visual, auditory, and tactile stimulation. Brain activity from three central areas (Cz, Pz, and Fz) was recorded on QEEG. These midline locations were chosen, as they are more reliable and less variable when compared to other periphery represented sites. Additionally, these midline sites overlie sensorimotor cortical regions of LE [36]. Results showed more improvement of motor skill acquisition in the experimental group (GII), as compared to the control group (GI), reflected clinically in increased scores of FIM and QEEG motor area excitability.

FIM was used in the current study, as it is a well-known, widely used assessment tool in rehabilitation, besides being valid and reliable for measuring any changes [37]. Both groups showed improvement in functional performance measured by FIM after receiving somatosensory and standard protocols of treatment, respectively, with more improvement in GII.

Regarding QEEG and despite significant improvement recorded in the three sites of QEEG in GII, that improvement was not significant when compared to GI. The control group (GI) showed improvement in area Cz only post-treatment, which may be due to the fact that those patients did not receive somatosensory training. The current results prove that adding somatosensory stimulation, in the form of augmented TS, to standard rehabilitation protocols improves motor (Cz), sensory (Pz), and learning (Fz) cortical mapping and facilitates neural plasticity in chronic stroke patients. However, this improvement was not sufficient to produce a notable or significant effect in brain plasticity. That might be attributed to duration of treatment in the current study (eight weeks) or the chronicity of the cases. The amplitude of motor evoked potential in the central zone (Cz) after LE training increased in both groups, which reflects short-term cortical adaptations correlated with motor learning. Motor maps expansions relate to better functional recovery in stroke patients [38].

It was reported that acute stroke patients treated with TS have sustained functional improvements up to three months, but improvements’ benefits disappeared by six-month and one-year follow-ups [26]. To the best of the research team’s knowledge, only seven clinical trials investigating the effects of TS on stroke patients were conducted until present [19,22,23,24,25,26,27], with three only being on LE function [23,24,25,26]. One study was carried out on acute stroke patients [23], while the other two studies used functional outcome measures only. More and above, all of them used a different technique of TS and evaluation, but all agreed with the findings of the current study. These findings can be attributed to the potential effect of adding sensory inputs on motor function. TS also facilitates sensory motor interaction through its multiple neural pathways of hot and cold sensation, activating several brain areas and enhancing experience-dependent organization of brain synapses and neural plasticity [22,39], hence stimulating neurotrophic factors and nerve growth factors involved with neural plasticity [40].

Bailey et al., 2008 also reported that the percentage of change of EEG during rest in different environments (normal or hot) was increased in the ratio of alpha to beta frequency, which might explain the effect of TS on cortical activities [41]. Adding pedaling cycling training to TS might have increased this effect, as it was reported that bipedal tasks require greater cortical control and inter limb coordination. Trans-callosal connections between homologous motor regions promote inter-hemispheric facilitation and communication that might contribute to enhanced excitability [42].

It was reported that sensory stimulation, using external tactile for a duration of three hours, applied once, improved tactile acuity in old adults and does not necessitate attention or active participation of the subjects [43]. However, activated brain areas by TS, whether hot or cold, are bigger than those stimulated by tactile or mechanical stimuli and is nearly the same as that of motor task [23]. Visual and auditory stimuli give more feedback to the brain, promote arousal, and prevent wrong motor learning. It was mentioned that even observing another person’s movement can recruit neural motor structure [44]. Measured by functional magnetic resonance imaging (fMRI), motor recovery of stroke patients has been improved after action observation, by reactivating the mirror neuron system and the action observation/action execution matching system, suggesting its key role in motor recovery and learning [45].

### Limitations

In the current study, adding somatosensory training to traditional physical therapy program resulted in decreasing disability and improving motor performance and cortical activity in stroke patients. However, these results are limited to the criteria of the selected sample with its statistical sequel.

Further studies are required on a bigger sample size and for longer durations of treatment. Additionally, comparing the effects of TS with other somatosensory modalities by using other functional and objective methods of evaluation is required. The current results show hope and interesting trends on the effects of somatosensory rehabilitation on chronic stroke patients, considering the deficiency of studies performed in this area.

## 5. Conclusions

TS is one of the advanced approaches in rehabilitation. Compared to the robotic-aided system, virtual reality, or other advanced strategies, TS is a simple, easy, low-cost modality, and it can be manufactured by using homemade tools, for example, water packs in developing countries. The current somatosensory approach has used augmented TS by adding tactile stimulation and managed to improve functional performance of the affected LE and neural activity of the brain.

## Figures and Tables

**Table 1:** Patient characteristics in the control group (GI) and intervention group (GII).

Patient Characteristics	Mean ± SD		MD	*t*-Value	*p*-Value
	Experimental Group	Control Group			
Age (years)	51.26 ± 2.71	50.86 ± 3.04	0.4	0.38	0.7
BMI (kg/m^2^)	28.26 ± 2.12	27.32 ± 2.21	0.94	1.19	0.24
Duration of illness (months)	13 ± 4.08	12.6 ± 4.03	0.4	0.27	078
Sex (Males/females)	10/5	9/6		(χ^2^ = 0.14)	0.7
Side of affection (Right/Left)	8/7	8/7		(χ^2^ = 0.13)	0.71

body mass index, BMI; SD, standard deviation; MD, mean difference; χ^2^, Chi-squared value; *p*-value, probability value.

**Table 2:** Functional Independence Measure (FIM), Cz-AR, Pz-AR and Fz-AR scores before and after treatment in GI and GII.

Clinical Evaluation	Experimental Group	Control Group		
	Mean ± SD	Mean ± SD	MD (95% CI)	*p*-Value
FIM				
Pretreatment	29.26 ± 1.86	29.2 ± 1.61	0.06 (−1.23: 1.37)	0.91
Post-treatment	33.26 ± 1.27	31.2 ± 1.78	2.06 (0.9: 3.22)	0.001
MD (95% CI)	−4 (−4.78: −3.21)	−2 (−2.78: −1.21)		
% of change	13.67	6.84		
	*p* = 0.001	*p* = 0.001		
Cz-AR				
Pretreatment	3.75 ± 0.37	3.86 ± 0.42	−0.11 (−0.4: 0.19)	0.46
Post-treatment	4.38 ± 0.41	4.19 ± 0.44	0.19 (−0.13: 0.5)	0.24
MD (95% CI)	−0.63 (−0.82: −0.43)	−0.33 (−0.52: −0.13)		
% of change	16.8	8.54		
	*p* = 0.001	*p* = 0.001		
Pz-AR				
Pretreatment	3.85 ± 0.49	3.95 ± 0.51	−0.1 (−0.47: 0.28)	0.61
Post-treatment	4.22 ± 0.37	4 ± 0.41	0.22 (−0.06: 0.52)	0.12
MD (95% CI)	−0.37 (−0.6: −0.13)	−0.05 (−0.28: 0.19)		
% of change	9.61	1.26		
	*p* = 0.004	*p* = 0.71		
Fz-AR				
Pretreatment	3.77 ± 0.47	3.8 ± 0.44	−0.03 (−0.36: 0.33)	0.93
Post-treatment	4.13 ± 0.41	3.9 ± 0.45	0.23 (−0.09: 0.55)	0.16
MD (95% CI)	−0.36 (−0.56: −0.13)	−0.1 (−0.32: 0.1)		
% of change	9.54	2.63		
	*p* = 0.002	*p* = 0.31		

SD, standard deviation; MD, mean difference; CI, confidence interval; *p*-value, level of significance.

## Data Availability

The data presented in this study are available on request from the corresponding author.
